# Expression of brown-midrib in a spontaneous sorghum mutant is linked to a 5′-UTR deletion in lignin biosynthesis gene *SbCAD2*

**DOI:** 10.1038/s41598-017-10119-1

**Published:** 2017-09-15

**Authors:** Huang Li, Yinghua Huang

**Affiliations:** 1 Department of Plant Biology, Ecology and Evolution, Oklahoma State University, Stillwater, OK 74078 USA; 2 United States Department of Agriculture - Agricultural Research Service (USDA-ARS), Plant Science Research Laboratory, Stillwater, OK 74075 USA

## Abstract

Brown midrib (bmr) mutants in sorghum (*Sorghum bicolor* (L.) Moench) and several other C4 grasses are associated with reduced lignin concentration, altered lignin composition and improved cell wall digestibility, which are desirable properties in biomass development for the emerging lignocellulosic biofuel industry. Studying bmr mutants has considerably expanded our understanding of the molecular basis underlying lignin biosynthesis and perturbation in grasses. In this study, we performed quantitative trait locus (QTL) analysis, identified and cloned a novel cinnamyl alcohol dehydrogenase allele (*SbCAD2*) that has an 8-bp deletion in its 5′-untranslated region (UTR), conferring the spontaneous brown midrib trait and lignin reduction in the sorghum germplasm line PI 595743. Complementation test and gene expression analysis revealed that this non-coding region alteration is associated with the significantly reduced expression of the *SbCAD2* in PI 595743 throughout its growth stages. Moreover, a promoter-GUS fusion study with transgenic *Arabidopsis thaliana* plants found that *SbCAD2* promoter is functionally conserved, driving a specific expression pattern in lignifying vascular tissues. Taken together, our results revealed the genetic basis of bmr occurrence in this spontaneous sorghum mutant and suggested the regulatory region of the *SbCAD2* can be a target site for optimizing lignin modification in sorghum and other bioenergy crops.

## Introduction

Sorghum is one of the premier biomass feedstocks for biofuel production because of its high biomass yield, outstanding drought tolerance and efficient nutrient usage^1^. In the renewable bioenergy industry, overcoming the intrinsic recalcitrance of biomass is critical for the development of lignocellulosic biofuel as a cost-effective alternative to fossil fuels^2^. Biomass recalcitrance is mainly due to the complex configuration of lignin polymer chains and the intertwined network of lignin and polysaccharides in plant cell walls^3^. In plants, the highly interwoven cell wall matrix rigidified by lignin is crucial for their structural integrity, water conduction and pathogen resistance^4, 5^. Hence, as a major impediment to plant biomass utilization and an essential component of the secondary cell wall, lignin has become a main research object in plant biology and its biosynthetic pathway has been extensively studied in the past several decades. In general, cell wall lignin is oxidatively polymerized primarily from three types of alcohols (monolignols), which are synthesized in cytosol from phenylalanine through successive deamination, reduction, hydroxylation and methylation steps^5^. Knowledge from the well-delineated monolignol biosynthetic pathway has led to the successful genetic manipulation of lignin content and composition in bioenergy grasses^6, 7^. Recently, in order to minimize developmental defects in lignin-modified plants, lignin modification strategy has evolved from simple perturbations of the general monolignol biosynthetic pathways to coordinated orchestrations of the lignin synthesis, deposition and integration into the cell wall network^8^. Therefore, a profound knowledge and advanced understanding of the underlying regulatory mechanisms of lignin production would benefit the long-term goal of manipulating cell wall structure in biomass feedstocks based on our needs.

Brown midrib (bmr) is a visual trait first documented in maize over eighty years ago and later mutagenesis-induced in sorghum and pearl millet^9–11^. In grasses, this trait is characterized by the reddish-brown pigment accumulated in leaf midribs and stems during the vegetative stage. In earlier years, this distinct pigmentation was found to be associated with higher ruminant digestion rate and thus became a desired feature in livestock forage development^12, 13^. Later, the property of reduced lignin content in bmr mutants was appreciated by plant geneticists and utilized for identifying candidate genes for manipulating the lignin production in grasses^14^. A number of mutagenized bmr mutants of sorghum have been characterized and the corresponding genes involved in specific enzymatic steps of monolignol biosynthetic pathway have been identified in recent years^15–18^. For instance, bmr6 mutant, originally generated from a diethyl sulfate mutagenesis sorghum population, was found to be associated with reduced cinnamyl alcohol dehydrogenase (CAD) activity^19^. CAD is a specialized enzyme of the alcohol dehydrogenase family involved in the conversion of the cinnamaldehydes into alcohols, the monomeric precursors of lignin. Downregulation of CAD genes generates atypical lignin polymers with the incorporation of phenolic aldehydes and becomes a promising strategy to improve cell wall digestibility^20–22^. Later on, a nonsense mutation in a sorghum *CAD* gene (*SbCAD2*) was identified to be responsible for the bmr6 phenotype. Specifically, the nonsense mutation truncates the coding region of *SbCAD2* prior to the conserved NADPH-binding and C-terminal domains and consequently, abolishes the function of the encoded protein^16, 17^.

Fiber digestion tests and field evaluation performed on sorghum varieties carrying the mutagenized bmr mutations found that they generally show higher digestibility and inferior agronomic performance relative to their wild-type counterparts, even though the effects of mutations are not uniformly expressed across different genetic backgrounds and are influenced by environmental conditions^14, 23^. Stacking different bmr mutations in sorghum has an additive effect on lignin content and cellulose-to-ethanol conversion^24^. Therefore, there are some recent efforts focusing on characterization of novel bmr sorghum lines derived from EMS (ethyl methanesulfonate)-mutagenized populations with the aim to incorporate new sources into the forage and biomass feedstock improvement^25, 26^. In summary, most sorghum bmr mutants studied so far are from mutagen-induced populations, and no spontaneous bmr phenotypes in sorghum have been characterized in detail at the molecular level.

In this study, we focused on PI 595743, a sorghum germplasm line showing a distinct bmr trait without any observable growth and developmental defects. Phenotypic evaluation confirmed the lignin deficiency in its brown vascular tissues. We next used QTL mapping, DNA sequencing and complementation test to identify that an 8-bp deletion in the 5′ UTR of *SbCAD2* is responsible for this novel spontaneous bmr phenotype. This deletion results in the down-regulation of *SbCAD2* expression in PI 595743 in a mechanism that is different from that underlying the reduced expression of *SbCAD2* in the bmr6 lines. Furthermore, we found that the function of *SbCAD2* promoter is conserved in both sorghum and Arabidopsis and this deletion region is implicated in the transcriptional regulation of *SbCAD2* expression in lignifying tissue. We expect that this alternative allele of *SbCAD2* gene will expand the repertoire of genetic resources for biomass improvement in sorghum, and more importantly, provide new insights into the complex underlying regulatory mechanism of the lignin biosynthetic pathway in grasses.

## Results

### Phenotypic characterization of the spontaneous bmr mutant

**Figure 1:**
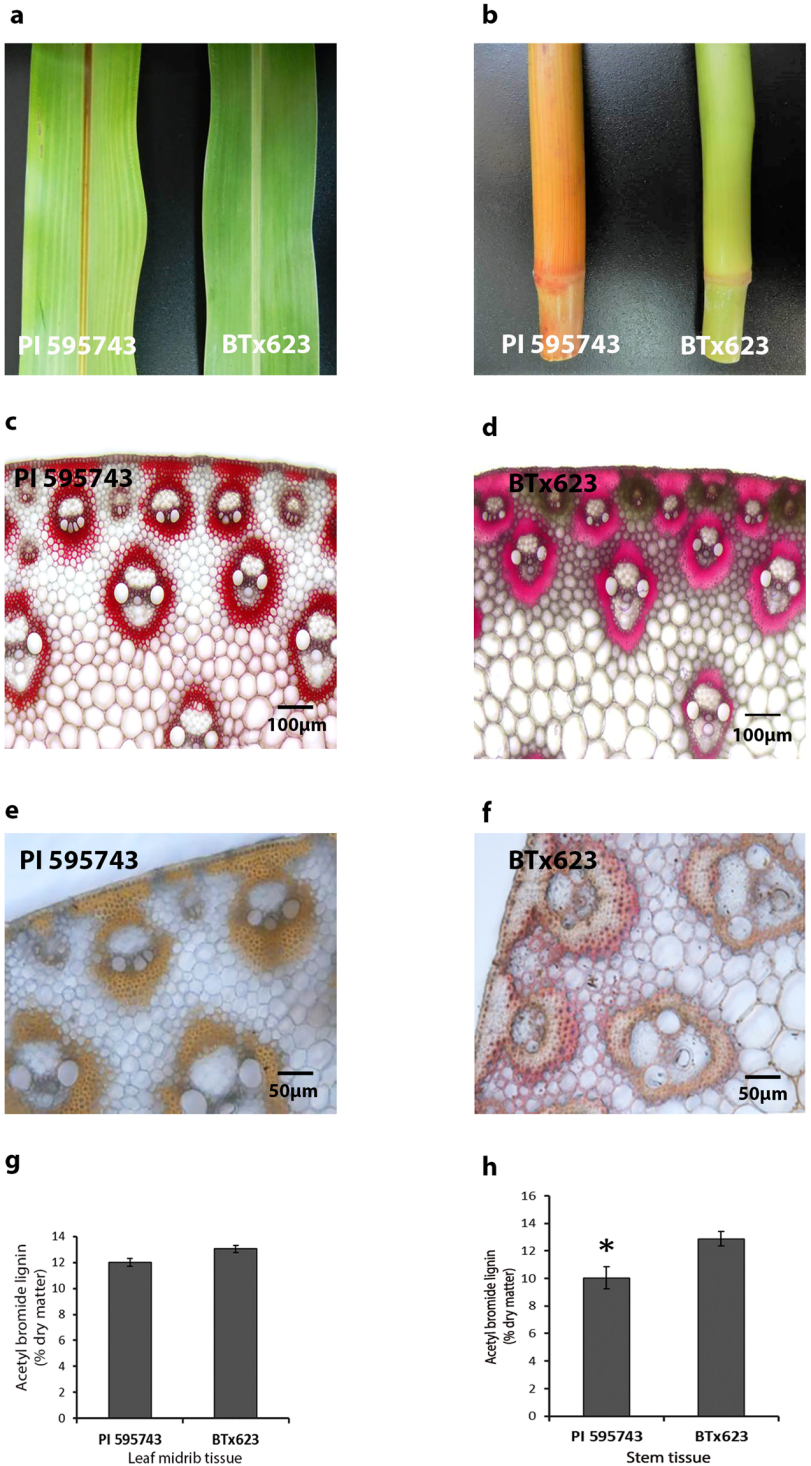
Phenotypic characterization of PI 595743 mutant. (**a**,**b)** Leaf midribs and stems of greenhouse-grown sorghum bmr mutant PI 595743 and non-bmr counterpart BTx623. (**c**,**d**) Phloroglucinol staining of cross sections of the second internodes above ground from PI 595743 and BTx623 plants. The microscopic images are representative of multiple experiments. (**e**,**f**) Maule staining of the samples used in the phloroglucinol staining. (**g**,**h**) Acetyl bromide lignin quantification of leaf midrib tissue and stems collected from PI 595743 and BTx623 lines at the end of vegetative stage. Each bar comprises the mean of three biological and three technical replicates. Error bars indicate the standard error from the mean. Asterisk indicates significant difference at 5% (P < 0.05) level of significance as determined by *t*-test.

Compared to the non-mutant sorghum plants with pale green midrib, PI 595743 first exhibits the characteristic reddish-brown color in the midrib at the 4, 5-leaf stage. The brown coloration accumulates with the expansion of the leaf blade and gradually fades as plants mature. The base of leaf on the abaxial side has the highest intensity of bmr color. Strong brown pigmentation was also observed throughout the stem of PI 595743 (Fig. 1a,b).

Phloglucinol-HCl staining revealed that the stem cross sections from PI 595743 and its non-bmr counterpart BTx623 displayed red color in lignified tissues, such as the subepidermal region and the scattered vascular bundles (Fig. 1c,d). However, the color contrast was different and PI 595743 had a more intense red coloration, indicating greater incorporation of cinnamaldehyde end-groups (hydroxycinnamyl aldehydes) in the lignin. This result is consistent with that of *CAD*-deficient mutants in Arabidopsis^27^. Moreover, when subjected to Maule staining, the pink-red coloration prominently presented in the BTx623 sclerenchyma cells surrounding vascular bundles was missed in the mutant samples (Fig. 1e,f), suggesting that the S unit content was below the detection limit of Maule stain. Together, these results suggested that the total lignin content, especially the composition of lignin, was altered in the vascular tissue of PI 595743. In addition to the histochemical staining, PI 595743 leaf midrib tissue and internode samples were analyzed by acetic bromide, a reagent for determining the total lignin concentration with spectrophotometry-based methods. The means of lignin concentrations of PI 595743 midrib tissue and stem at the end of the vegetative stage were 119.9 and 100.4 mg/g, respectively, representing an average 8% reduction compared to the BTx623 controls (Fig. 1g,h). These results provided quantitative evidence that lignin biosynthesis was indeed impaired in PI 595743 plants.

### QTL analysis of bmr trait in the RIL population

**Figure 2:**
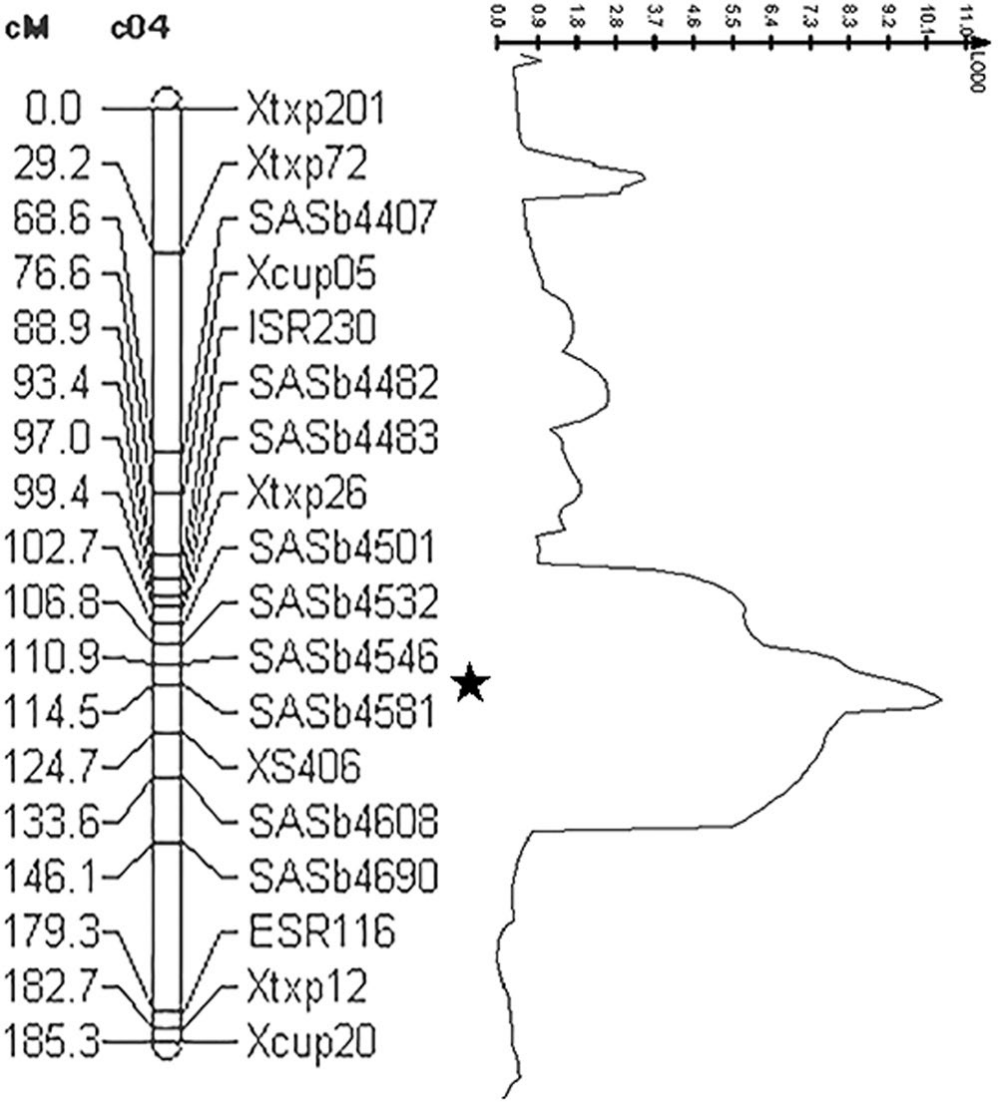
The major QTL underlying the bmr trait in PI 595743 identified in this study. The centiMorgan (cM) distances between marker loci on chromosome 4 are shown along the left and the positions of markers are shown to the right. The LOD (logarithm of the odds) score peak profile using CIM analysis indicates the LOD value for this QTL on chromosome 4 is 10.77.

**Figure 3:**
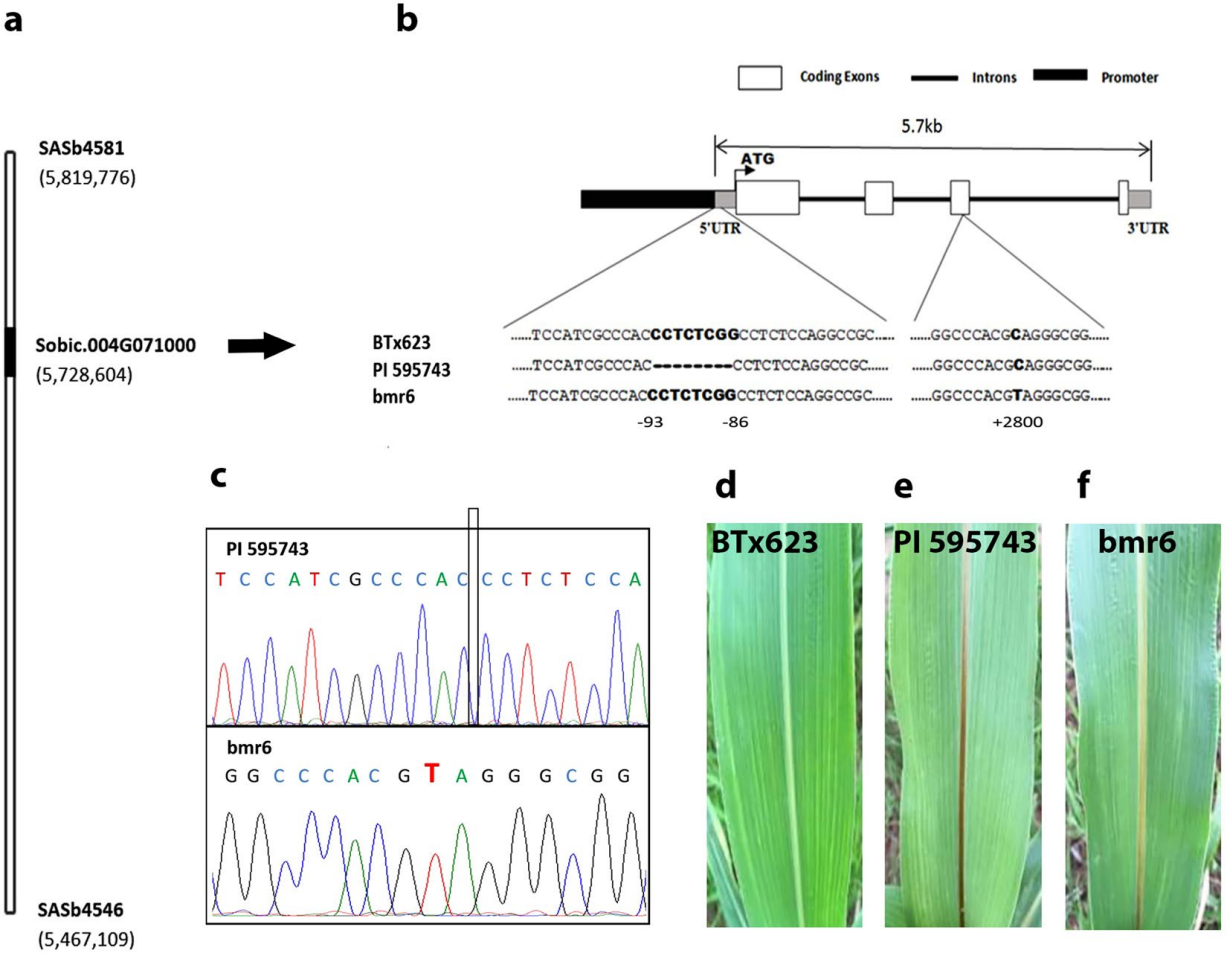
Map-based cloning of the *SbCAD2*. (**a**) Physical map of the target gene flanked by two microsatellite markers with an interval. Target gene *Sobic.004G071000* (*SbCAD2*) is shown in bold. The base pair numbers correspond to the sorghum genome database version 3.1.1 (www.phytozome.net/sorghum). (**b**) Schematic representation of the *SbCAD2* structure and sequence comparison of the *SbCAD2* in BTx623, PI 595743 and bmr6. Numbers indicate distances from the start codon. (**c**) Sequence differences are confirmed by DNA sequencing. Box indicates the 8-bp deletion region in PI 595743. C to T transition in bmr6 is highlighted in red. (**d**–**f**) The differences of leaf midrib coloration are observed among BTx623, PI 595743 and bmr6 lines.

The visual brown coloration in bmr mutants has made this trait an ideal candidate for QTL study to reveal the genetic basis of altered lignification. During the process of mapping population development, we found that the F_1_ plants derived from a cross between the spontaneous mutant PI 595743 and BTx623 showed a non-bmr phenotype, and the F_2_ progeny revealed a 3:1 Mendelian-like segregation, suggesting that this spontaneous phenotype is controlled by a single recessive gene. This result is consistent with previous findings that brown midrib in grasses is a recessive trait caused by single-gene disruption in the lignin biosynthetic pathway^14, 28^. Our QTL mapping study integrated with the sequenced BTx623 genome and previously published maps contributed to the rapid development of novel markers within an initially identified QTL interval and the improvement of map resolution. At the end, we constructed a genetic linkage map spanning all ten chromosomes with a marker-saturated chromosome 4 (Fig. S1). The primer sequence information of new SSR markers developed for this study is given in Table S1. After several rounds of iterative QTL analysis, a major QTL explaining 71.2% of the phenotypic variation was determined to be in the interval of markers SASb4546 and SASb4581, with a genetic distance of 2.5 cM from SASb4546 and 1.1 cM from SASb4581 (Fig. 2). This 3.6 cM interval corresponds to 353 kb in terms of physical distance on the sorghum genome sequence (Fig. 3a).

### Candidate gene selection and validation

Combining the mapping information with sorghum genome sequence database, forty-eight genes have been localized in the region defined by markers SASb4546 and SASb4581. These genes, together with their locations, lengths and functional annotations, are given in Table S2. When we focused on searching for the genes that share functional homology with characterized proteins related to lignin biosynthesis and plant cell wall formation, we found that *Sobic.004g071000*, the gene encoding a cinnamyl alcohol dehydrogenase (CAD) protein that catalyzes the last step of monolignol biosynthetic pathway, was present in this region. In addition, *Sobic.004g071000* (*SbCAD2*) had been previously identified as the gene underlying the mutagenized bmr6 phenotype in sorghum. Thus, we selected this gene as a promising candidate for further study. Moreover, this selection was supported by the result of our previous phloroglucinol staining which indicates more aldehyde groups in PI 595743 tissues.

A complementation test was performed by crossing PI 595743 with the bmr6 mutant line to determine whether these two mutants are caused by the same gene or two different genes. All twenty-five F_1_ progeny plants showed the characteristic brown color in their midribs, suggesting these two mutated phenotypes cannot be complemented to each other and they could be either the same mutant alleles, or different mutant alleles of the same gene.

In comparison to the BTx623 reference genome, PI 595743 showed no sequence difference in the exons and introns of *SbCAD2*. However, an 8-bp nucleotide deletion (CCTCTCGG) was found within its 5′-untranslated region located 93 bp upstream of the start codon (Fig. 3b,c). Furthermore, we designed a specific pair of primers based on this deletion difference and used it to genotype each line in the RIL population. This newly developed maker was mapped to a location that defined the peak of the QTL on chromosome 4 as expected. We also found that this 8-bp deletion co-segregates with the bmr phenotype in the RIL population, which provides further evidence that *SbCAD2* is the gene responsible for the expression of bmr trait in the spontaneous mutant PI 595743.

### Expression analysis of the *SbCAD2*

**Figure 4:**
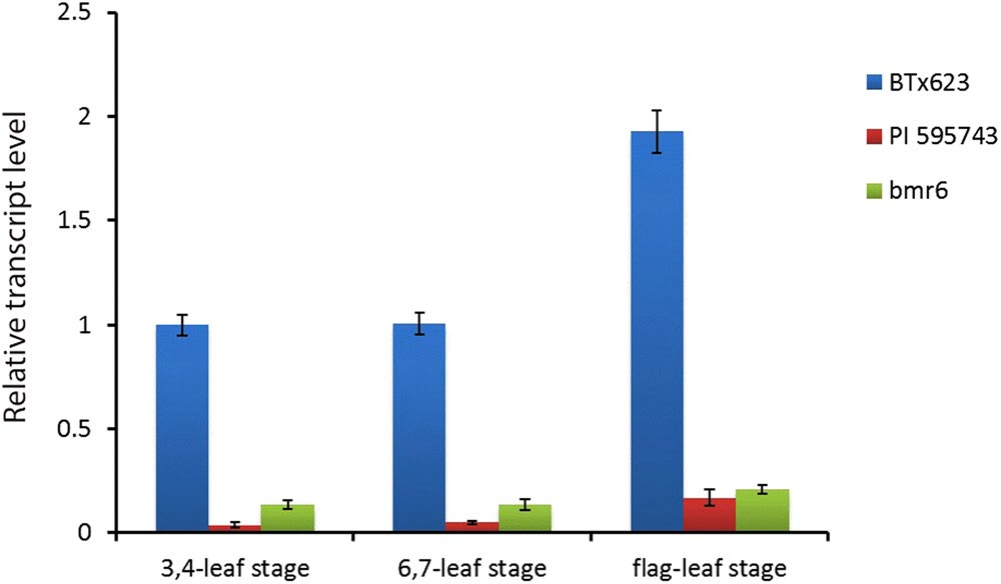
Expression of the *SbCAD2* gene at three different developmental stages. Relative expression of *SbCAD2* was normalized by geometric means of the housekeeping gene (see Methods). Data are shown as mean ± S.D.

Real-time quantitative PCR (qRT-PCR) was performed to investigate the *SbCAD2* expression pattern throughout three representative developmental stages in PI 595743 and the non-bmr control BTx623. Considering the fact that allelic variation in the *SbCAD2* between PI 595743 and bmr6 could also affect the expression pattern, along with the observed difference of brown color intensity, we included the bmr6 plants in the gene expression study. Consistent with its role in lignin biosynthesis and cell wall rigidification, transcript levels of *SbCAD2* accumulate along with the development of all plants. While compared to the non-bmr line, the accumulation of *SbCAD2* transcripts is significantly reduced in both bmr mutants at all three stages. Interestingly, the *SbCAD2* expression was further decreased in PI 595743 relative to the bmr6. At the end of the vegetative stage, the *SbCAD2* expression was approximately 20-fold lower in PI 595743 relative to the non-bmr counterpart (Fig. 4).

### Analysis of the *SbCAD2* promoter in transgenic Arabidopsis

**Figure 5:**
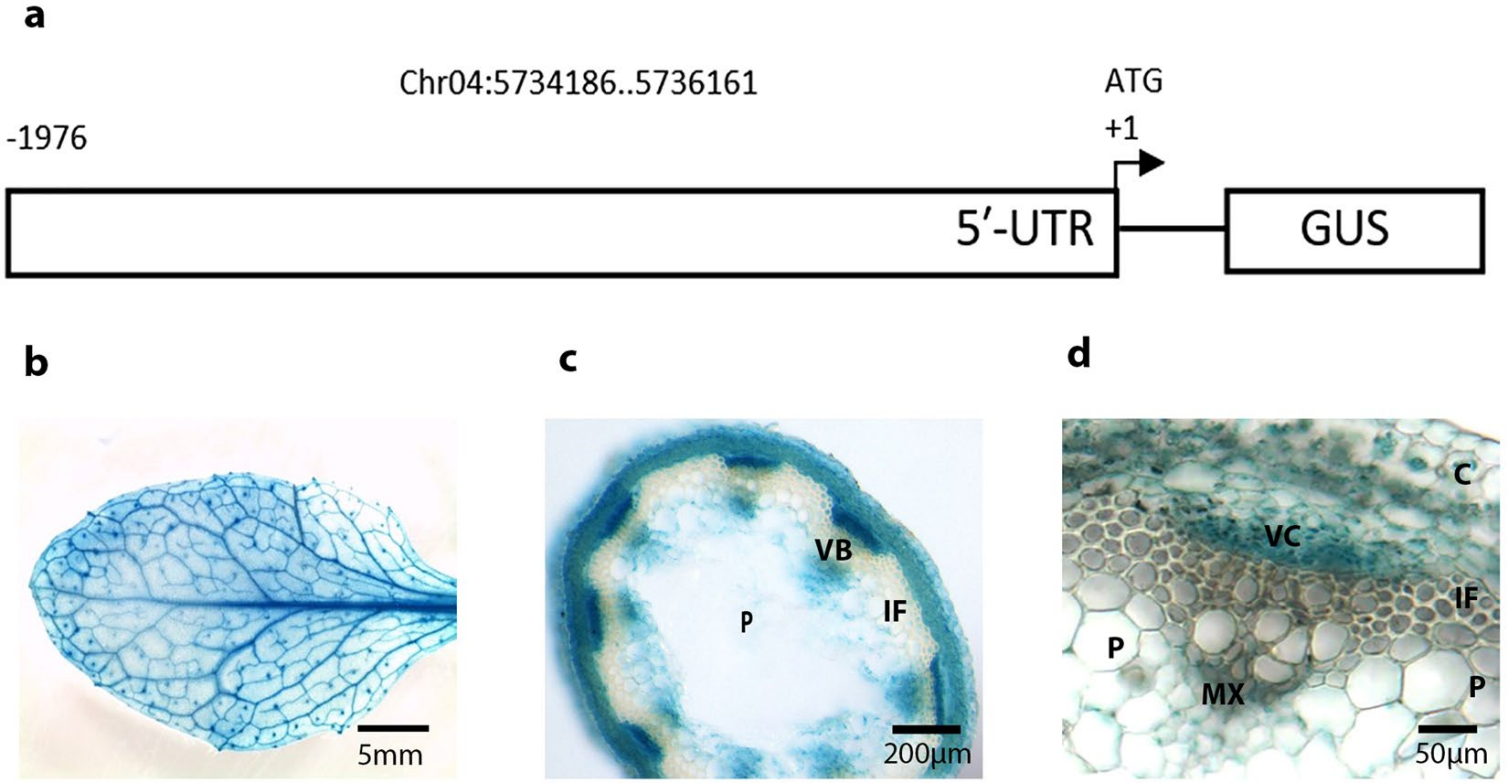
Histochemical analysis of GUS activity driven by the full-length *SbCAD2* in transgenic Arabidopsis plants. (**a**) Schematic representation of the *SbCAD2* promoter-GUS fusion construct. ‘ATG’ indicates the initiation codon site. Numbers indicate the physical position of full promoter sequence. (**b**–**d**) *SbCAD2* promoter activity is revealed by blue coloration indicating GUS activity in that region. (**b**) General view of a fully expanded leaf. (**c**) Cross section of the lower part of inflorescence stem. (**d**) Enlargement of a vascular bundle, cambial and xylem zone. P, pith; C, cortex; VC, vascular cambium; MX, metaxylem; VB, vascular bundle; IF, interfascicular fibers. All images were obtained from the representatives of 8-week-old independent transgenic lines.

To determine if the *SbCAD2* promoter of non-bmr line BTx623 is active in Arabidopsis and its expression pattern is conserved in the lignifying vascular tissues, transgenic Arabidopsis plants containing a p*SbCAD2*::GUS construct were developed (Fig. 5a) and the expression pattern directed by p*SbCAD2* was examined. In fully-expanded leaves, the *SbCAD2* promoter directed high specific GUS activity in the midrib and vascular veins (Fig. 5b). Cross sections of the mature inflorescence stems revealed that GUS activity was strongly associated with the developing xylem and the cambial regions adjacent to the metaxylem. No GUS activity was observed in the pith or in the cortex region (Fig. 5c,d). Thus, these data correspond to the function of *SbCAD2* and suggest that the mechanism controlling developmental expression of the *SbCAD2* promoter in lignifying tissues is conserved among grasses and dicots.

## Discussion

Developing brown midrib mutant lines and identifying the genetic basis of lignin deficiency in these mutants are of increased interest due to their potential to reduce biomass recalcitrance. There are more than ten enzymatic steps involved in the monolignol biosynthetic pathway, leading to the synthesis of monolignol precursors for lignin polymerization^5, 29^. Theoretically speaking, any perturbation in one of these steps could affect the lignin production. To date, only a few steps of the monolignol pathway were found to be directly involved in the exhibition of bmr in sorghum^15, 16, 30^ and most sorghum bmr mutants developed from mutagenesis are allelic^25, 30^. Hence, our main objective was to characterize a naturally-occurring bmr mutant in sorghum, with an attempt to isolate novel bmr loci for further study. Interestingly, the causal gene underlying the spontaneous bmr mutant in this study was identified to be *SbCAD2*, the same gene responsible for the bmr6 phenotype. This high occurrence of allelic bmr mutants could be simply ascribed to the formation of brown color itself. In other words, disruption of a particular step in the monolignol biosynthetic pathway may not be sufficient to induce the generation of a specific bmr phenotype. While the exact cause of brown pigmentation in lignified tissue is elusive, the bmr trait in mutants or transgenic lines with impaired CAD activity has been attributed to the incorporation of cinnamyl aldehydes into lignin in place of cinnamyl alcohols^14^. This fact is consistent with the phloroglucinol staining result we observed in PI 595743. Another possible explanation of the discrepancy between bmr occurrence and lignin disruption is that modifying some enzymatic steps in the monolignol biosynthetic pathway could result in relatively more severe side-effects on plant growth and development. This hypothesis is supported by several previous studies on lignin-modified mutants. For example, Arabidopsis plants defective in C3H (*p*-coumarate 3-hydroxylase), the second enzyme in the monolignol pathway, exhibit severe dwarfism and sterility^31^. Similar deleterious defects were also observed in the diethyl sulfate (DES)-induced bmr lines of sorghum, precluding the subsequent genetic study on those mutants^9^.

Due to the importance and plasticity of the last step of monolignol pathway, the impacts of CAD disruption on cell wall properties have been intensively studied in several plant species. CAD-deficient mutants^32, 33^ and transgenic lines with down-regulated CAD activity^34, 35^ were generally associated with reduced lignin content and/or altered lignin composition change, even though there were some reported inconsistencies partly due to functional redundancy of CAD paralogs or incomplete disruption of CAD expression. In sorghum, the CAD-deficient bmr6 mutant showed reductions in lignin content, altered lignin composition and an improved cell wall digestibility^13, 24^. Despite the substantial impact on lignin biosynthesis, agronomic evaluation found that the bmr6 mutant was not associated with significant negative impacts on plant fitness in comparison with its near-isogenic counterpart^25^. In the current work, a spontaneous sorghum bmr mutant, PI 595743, showed a modest decrease in lignin content and dramatic change of lignin composition in certain lignified cell types, in agreement with the aforementioned fact that monolignol biosynthesis is highly plastic as to allow CAD-deficient plants to form lignin polymers directly from cinnamyl aldehydes. The accumulation of aldehydes and reduction of the S lignin subunit in PI 595743 were revealed by phloroglucinol staining and the Maule staining, respectively. These histochemical results provide the first clue that the CAD enzymatic activity is likely affected in PI 595743. Gene expression assay further confirmed the down-regulation of *SbCAD2* gene during the development of PI 595743 plants. The significant reduction of CAD activity due to a nonsense mutation was reported in bmr6^17^. Based on the allelic variation, it may prove of interest to examine to what extent the CAD activity is impaired in PI 595743. Furthermore, comparative evaluation of different CAD mutants under the same genetic background, for example, within the near-isogenic lines would be a good starting point to study whether genetic variants in the *SbCAD2* gene have different effects on lignin composition, biomass digestibility and the overall agronomic performance.

Previous research on the bmr6 mutant revealed that a nonsense mutation-mediated mRNA decay mechanism was very likely involved in the downregulation of *SbCAD2* in its vascular tissues^17^. In the present work, based on collective evidences from histochemical analysis, QTL mapping, association study and complementation test, an 8-bp deletion in the 5′ UTR of *SbCAD2* was linked to the dark-brown pigmentation and the significant reduction of *SbCAD2* expression in the spontaneous mutant PI 595743. Thus, an obvious question from these results is: how does this deletion in an untranslated region affect the expression of *SbCAD2* and lead to a mutated phenotype? As a fundamental structural and regulatory region of eukaryotic genes, 5′ UTR mainly plays a role in the regulation of mRNA translation, providing a novel layer of coordinated control of gene expression^36, 37^. Parts of the 5′ UTR may also contain regulatory elements and may be part of the promoter. 5′ UTR-mediated regulation of transcript abundance and translational efficiency has been reported in plant development and stress responses^38–40^. In the case of PI 595743, the 8-bp deletion within the 5′ UTR could affect its qualitative features, such as length, GC content and potential for secondary structure formation, associated with the translational regulation. There is also a possibility that this deletion is involved in the transcriptional regulation of *SbCAD2* by interfering with potential binding sites, an interaction mechanism totally different from the mRNA decay mechanism underlying the reduced expression of *SbCAD2* in the bmr6 mutant. Further studies on heterologous expression of a reporter gene fused with this mutated 5′ UTR or promoter deletion analysis are needed to dissect the association between this specific deletion and lignin pathway perturbation.

The active expression of GUS gene in the lignifying tissue of Arabidopsis plants driven by the promoter of the sorghum *CAD2* gene reflects the conservation of mechanism that underlies the transcriptional regulation of monolignol biosynthetic pathway in higher plants. The conservative property of lignin biosynthesis in plants has been investigated and discussed previously from different perspectives^41–43^. Evolutionarily speaking, the signals controlling vascular expression of lignin biosynthetic genes are more likely to be highly conserved in higher plants since lignin biosynthesis is an early adaptation feature for vascular plants to survive in the terrestrial environment^41, 44^. In addition, the maintenance of conserved motifs in many lignin biosynthetic genes suggested a common catalytic mechanism might be shared during the process of lignification in plants^45^.

Lignin biosynthesis is a highly coordinated process that involves the expression of multiple monolignol genes and the coordinated regulation of transcription factors^44, 46^. Knowledge gained from this study could open an avenue for us to decipher the regulatory aspects of the *SbCAD2* gene and reveal the occurrence of bmr phenotype and lignin production in grasses at the transcription and post-transcription level.

## Methods

### Plant materials and mapping population

The plant materials used in this study included the spontaneous sorghum bmr mutant (PI 595743), the elite inbred line (BTx623) and the bmr6 mutant. PI 595743 is a photoperiod insensitive conversion of SC 1201 selected from the World Sorghum Collection. It was developed in Texas, United States and used as a breeding material because of its early maturing, enhanced height and forage potential. In our study, an F_6_ recombinant inbred line (RIL) population was developed by crossing BTx623 and PI 595743. Specifically, PI 595743 (pollen donor) was crossed with BTx623 (manually emasculated) to obtain heterozygous F_1_ seeds, and the F_1_ plants were self-pollinated to produce the F_2_ segregating population. A set of 188 F_6_ RILs were obtained from the F_2_ family by the single-seed descent method.

### Lignin detection and quantification

Stem internodes collected from two developmentally-equivalent parental lines were hand-sectioned to a thickness of 200 μm using double-edge razor blades. Phloroglucinol stains the coniferylaldehyde end-groups present in lignin, forming a red-pink color in acidic conditions^47^. Briefly, sections were stained with phloroglucinol-HCl solution consisting of 1.0% (w/v) phloroglucinol, 3.7% (w/v) HCl, and 10% (v/v) ethanol for 1 min and mounted onto microscope slides for observation. Maule reagent was used to examine the sinapyl alcohol monomers (S lignin) content and localization in vascular tissues as described previously^48^. Sections were prefixed in 4% glutaraldehyde for 1 h and rinsed with sterile water. After that, sections were immersed in 1% (w/v) potassium permanganate solution for 5 min followed by a water rinse. They were then treated with 10% HCl (w/v) for 5 min, rinsed with water, and mounted on microscope slides in concentrated NH_4_OH. Stained sections were immediately observed under bright-field lighting on a Nikon 80i microscope and photographed.

For total lignin quantification, stem and midrib materials collected from PI 595743 and BTx623 plants at the end of vegetative stages were dried and pulverized into powder with metal beads in a vibratory solid sample homogenizer. Acetyl bromide (AcBr)-soluble lignin was quantified using the reported procedure^49, 50^. Briefly, triplicate samples of 10 mg powder from stem and midrib tissues were sequentially washed with 500 μL of water, ethanol, chloroform and acetone for 30 min, respectively. Washed samples were placed in glass 20 mL scintillation vials and heated overnight at 70 °C. Two and a half microliters of 25% AcBr solution was added to each vial and incubated at 50 °C for 2 h with occasional swirling. After cooling to room temperature, each sample was transferred into a 50 mL volumetric flask holding 10 mL of 2 N sodium hydroxide, and 12 mL of acetic acid. More acetic acid was used to bring the solution to the fixed volume. Samples were settled at room temperature overnight and the absorbance of the resultant solution at 280 nm was determined using a spectrophotometer. The lignin percentage of the dry sample was calculated from the regression equation of lignin content in herbaceous samples^51^.

### QTL mapping

Phenotyping was conducted around the time when the brown pigmentation in midrib reaches its maximum intensity, approximately 6 weeks after the planting. The leaf midrib phenotype of each RIL was visually scored and confirmed by the data from three replicates.

For genotyping, genomic DNA was extracted from seedlings of two parental lines and each F_6_ RIL using a modified CTAB protocol^52^. Initially, 1019 simple sequence repeat (SSR) markers in sorghum were used to survey their polymorphisms between BTx623 and PI 595743. Among them, 121 were identified as polymorphic and used for genotyping the entire population. PCR products were analyzed by ABI 3730xl DNA Analyzer (Applied Biosystems) and scored using GeneMarker version 4.0 (Soft Genetics LLC).

A linkage map was constructed by MAPMAKER/Exp 3.0^53^ with a logarithm of odds score (LOD) of 3.0 and maximum linkage threshold of 40 cM. The recombination frequency between linked loci was transformed into genetic distance (centimorgan, cM) using the Kosambi’s function. The output files were imported into QTL Cartographer version 2.5^53^ for QTL analysis. Single Marker Analysis, Composite Interval Mapping (CIM) and Multiple Interval Mapping (MIM) analyses were performed by following the lab procedure^52^.

### Candidate gene selection

After the major QTL was localized, the nucleotide sequences of two markers flanking this region were used in BLAST searches against sorghum genome database version 3.1.1 (www.phytozome.net/sorghum) to define the start and end position of the physical interval. All genes within this interval were subject to functional annotation and protein homology detection. A gene was identified as a real candidate only if there was experimental evidence that it is involved with brown pigmentation in vascular tissue, lignin biosynthesis or secondary cell wall formation.

### Complementation test and sequencing

Complementation crosses were carried out by emasculating the bmr6 plants at the floral stage followed by hand pollination with PI 595743 pollen. Flowers were bagged to prevent contamination. Seeds were harvested from mature plants and planted in green house for phenotypic evaluation.

Approximately 2 kb of upstream sequence immediately before the coding start codon and the 5.7 kb genomic sequence of *SbCAD2* (*Sobic.004G071000*) in PI 595743 were sequenced as overlapping fragments generated by high-fidelity PCR amplification. Primer information is given in Table S3. The corresponding sequences from BTx623 were retrieved from the sorghum genome database as the reference for comparison.

### qRT-PCR analysis

To analyze the expression pattern of *SbCAD2* featured in the spontaneous bmr mutant PI 595743, non-bmr BTx623 and bmr6, samples were prepared from each line at the 3,4-leaf stage, the 6, 7-leaf stage and near the end of vegetative stage (emergence of the flag leaf). Fresh leaf tissues from the same location in each plant were harvested, and immediately ground in liquid nitrogen. Total RNA was isolated using TRIzol reagent (Invitrogen) following the manufacturer’s instructions, and 1 μg aliquots were treated with DNase and then reverse transcribed. The transcriptional profile was analyzed by qRT-PCR using the SYBR Green PCR Master Mix (Applied Biosystems) with the Biorad Real-Time PCR System. Primer specificity was verified by cloning and sequencing PCR products obtained from BTx623 cDNA. The melting curve analysis and the evaluation of primer amplification efficiency were performed before running the cDNA samples. Relative expression levels were calculated from the cycle threshold using 2^−ΔΔCt^ method^54^. A sorghum *β-tubulin* gene (*Sobic.002g350400*) was used as the internal control. The data were the average of three biological replicates. Primers used for qRT-PCR are listed in Table S3.

### Generation of a *SbCAD2* promoter-GUS fusion construct

In order to explore the function of *SbCAD2* promoter in lignifying tissue and establish a platform for studying the regulatory mechanism underlying *SbCAD2* expression, a construct of the full length *SbCAD2* promoter (p*SbCAD2*) fused to the β-glucuronidase (GUS) reporter gene (uidA) was generated. Specifically, the full length promoter (1976 bp of upstream sequence before the ATG) was PCR amplified from BTx623 genomic DNA by using region-specific primers and the high-fidelity polymerase mix. The PCR product was purified and cloned into the promoterless GUS vector pBI101, producing a fusion construct by following the manual instructions of the In-Fusion HD Cloning Kits (Clontech Laboratories, Inc.). The insertion was PCR amplified and confirmed by sequencing before transformation.

### Arabidopsis transformation and GUS activity localization

The full-length promoter-GUS fusion construct was introduced into *Agrobacterium tumefaciens* strain LBA4404 using the freezing/heat shock method. Transgenic Arabidopsis plants carrying the p*SbCAD2::GUS* expression cassette were generated by *Agrobacterium*-mediated transformation using the floral dip method^55, 56^. In order to confirm the specific expression conferred by the *SbCAD2* promoter, a transformation using a CaMV 35 S::GUS construct (pBI 121) was performed in parallel.

Histochemical location of GUS activities in transgenic T_1_ and T_2_ plants from different lines was investigated as described in the protocol^57^. Briefly, the basal region of the floral stems from each equivalently developed plants were collected and incubated for 12–24 h at 37 °C in GUS staining solution: 100 mM sodium phosphate (pH 7), 5 mM EDTA (pH 8), 0.5 mM ferrocyanide, 0.5 mM ferricyanide, 0.5 mg/mL X-Gluc (5-bromo-4-chloro-3-indolyl-β-d-glucuronide). A series of ethanol with different concentration (50%, 75%, and 95%) were used to replace the staining solution and remove chloroplast pigments. Crossed sections (80–150 μm) were observed for blue pigment deposition with a Nikon 80i microscope and photographed with an affiliated CCD camera.

## Electronic supplementary material

Supplementary Information
