# Supplementary material for: Expression of brown-midrib in a spontaneous sorghum mutant is linked to a 5′-UTR deletion in lignin biosynthesis gene SbCAD2
Source: Sci Rep. 2017 Sep 15;7:11664. doi: 10.1038/s41598-017-10119-1 (PMC5601950; doi:10.1038/s41598-017-10119-1)
Supplement: Supplementary file 1 — Supplementary Information [file 41598_2017_10119_MOESM1_ESM.doc]

**Expression of brown-midrib in a spontaneous sorghum mutant is linked to a 5′-UTR deletion in lignin biosynthesis gene *SbCAD2***

Huang Li and Yinghua Huang *


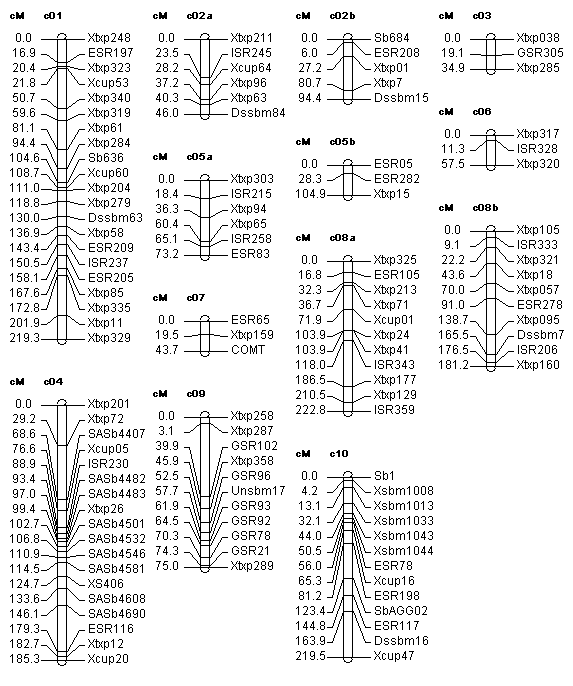


**Fig. S1 Genetic linkage map constructed from the BTx623 X PI 595743 recombinant inbred line population.**

**Table S1 List of novel microsatellite markers used for QTL mapping.**

| **Primer name** | **Forward sequence** | **Reverse sequence** | **SSR motif** | **Tm** | **Product size** |
| --- | --- | --- | --- | --- | --- |
| **SASb4348** | CTTGCGTTTATCCAGGAAGC | GGCTCATCATCATGTCAACG | (AT)36 | 60.0 | 240 |
| **SASb4407** | GACCTGGCCATGAAAAGAAA | CAATACCGTTGCAAGTCGTG | (AC)58(AG)23 | 60.1 | 282 |
| **SASb4451** | GCCACGGAAAAAGAAACTCA | TGTAGAATAGCGCTGCCGTA | (AT)17(AC)10 | 60.0 | 197 |
| **SASb4464** | TACGTGTTTCTCTGATGGCCTA | AGCTAGACCCGTTTGAATCTCTAT | (AT)52 | 59.0 | 351 |
| **SASb4465** | AGACCGCAAGTCAAAACTAAGC | AATGCCAACCTCATTTGCC | (TA)18 | 60.5 | 385 |
| **SASb4482a** | CACCATTTGTTTGCACCTCTTA | TTGACACTTACATGGCGAGATAG | (ATCT)11 | 59.7 | 385 |
| **SASb4482b** | AAATAACCCTCCCTCTGCATCT | AAATCTTCCCTTCTCCTCCTTG | (CT)34 | 60.1 | 291 |
| **SASb4487** | AACTAAAACCGGCAATAGCTCA | GGCCACAAAGTGACAGAATACA | (AT)39 | 60.0 | 386 |
| **SASb4496** | CGGCTGATGGAGAAGACTAAAC | GGATCACGAATGTCTAAAAGCC | (TC)20 | 60.0 | 350 |
| **SASb4501** | GCAGACACGTTACAAAGCATTC | AAGATGATAGCCACGTCAGGAT | (TAGA)18 | 59.9 | 359 |
| **SASb4502** | GCGCGTACACAGAAATATACGA | TCCATTCACCAATGAAAGCC | (CT)31 | 60.5 | 368 |
| **SASb4510** | ACGTTTTCGAGCTTTTGGATAC | TTTCTCCCTTCTTGATTTGGAA | (AT)12 | 59.8 | 291 |
| **SASb4516** | GTGACGATGGACTCACTGCTAC | CTCCTATGTGTATGTGCGTGCT | (TG)19 | 60.1 | 346 |
| **SASb4526** | TTGTGAAGGTTCCGTTCCTAAT | GGAGAGACGGTGAAATAGATGG | (TC)12 | 59.9 | 341 |
| **SASb4532** | AGACGGGACACACACACTTATG | CTCCATCTGGTTTCAAGGTTTC | (CT)20 | 60.1 | 380 |
| **SASb4536** | TTAGCAGGCTTCCAGGTTAAAA | GATCGCTCACGTAATAAGCAAGT | (TA)30 | 60.0 | 346 |
| **SASb4546** | TCTCTCATCTCTGAACTGTCGC | GAAAACTCGGAAAGACAACAGG | (CT)15 | 59.9 | 137 |
| **SASb4581** | CGTCAGGACGAGATGCACTA | ACAAATCCCTCCCCCATTAG | (GA)21 | 60.0 | 150 |
| **SASb4592** | GTTCGACAACGCGGAGTAAC | GCATCCCAACAGCTCCATAC | (AT)56 (AT)24 | 60.5 | 319 |
| **SASb4608** | AACCATGCATGAGAACCAAA | TGCAATGCCTACCTCCATTA | (GT)51 | 59.0 | 314 |
| **SASb4613** | CGGAGCTTCAAATGTGTTGT | CGATAGAATTTGCCGCAGTA | (AT)46 | 58.8 | 226 |
| **SASb4690** | TCGCGTCGATACAGGTAAAC | GGGTAAGCCACCCTCTCTCT | (GA)29 | 59.3 | 180 |
| **SASb4693** | CCACACAAAAATGGCTGAAC | TGTTTTCGGTCTCTCTGGAA | (AT)57 | 58.7 | 237 |
| **SASb4722** | GCCCACACCGTATAATCATC | AGTCAACATCCATTGCCTCA | (AT)28 (TG)34 | 58.3 | 302 |
| **SASb4726** | TGCTCACGATAGATGGCTTC | ATCACTGATCCAGCCACGTA | (TA)40 | 59.0 | 226 |
| **SASb4731** | CAGATCCACGCAGATTTCAA | GCACGTGCCTCTCTCTCTCT | (GA)26 | 59.8 | 205 |
| **SASb4775** | ACACATGTCGAAACGGATGA | CGTTACCAACAGCTTCAACG | (AT)47 | 59.6 | 192 |
| **SASb4804** | TGCTGAACTCAGCTTGATGC | CATCTTGCTGTCGTCCTTGA | (AT)29 | 60.1 | 304 |
| **SASb4811** | AAAAAGACGCTGTGCTCCAT | TGTTGGACTGTCACACCTTCA | (AT)43 | 60.0 | 310 |
| **SASb4817a** | GCTGGGTCCATGTCAACTACT | CCCCAGTGTCTCGCTTATTT | (AT)55 | 59.1 | 333 |
| **SASb4817b** | CAGGAAGAAAATTGGGTGGA | AAGAAGAGACCGCCCAAGAC | (AT)25 | 60.0 | 384 |
| **SASb4817c** | TCCACCAAGCTTCGTCTTCT | CAAATCATCCATGGCCAATC | (AT)37 | 60.5 | 269 |
| **SASb4844** | AGCAAAGCGAAGAAATGGAA | GATGGCTCACCATCACATCA | (ATCT)35 | 60.3 | 396 |

SASb stands for Stillwater-ARS (SA) Sorghum bicolor (Sb); the first number in the microsatellite name represents the chromosome number and the following three digits represent the physical position on that chromosome.

**Table S2 List of genes residing in the QTL interval and their putative function**.

| **Gene/marker ID** | **Location** | **Gene length (bp)** | **Gene annotation** |
| --- | --- | --- | --- |
| **SASb4546 marker** | 5466937...5467109 | NA | NA |
| ***Sobic.004G067200*** | 5479414..5480218 | 805 | similar to putative uncharacterized protein |
| ***Sobic.004G067300*** | 5487373..5494686 | 7314 | AP-3 complex subunit sigma |
| ***Sobic.004G067400*** | 5495211..5500051 | 4841 | unknown |
| ***Sobic.004G067500*** | 5502482..5510032 | 7551 | unknown |
| ***Sobic.004G067600*** | 5511765..5514381 | 2617 | unknown |
| ***Sobic.004G067700*** | 5515169..5519814 | 4646 | Zn-finger and ring finger domain |
| ***Sobic.004G067800*** | 5523980..5525565 | 1586 | putative zinc-binding protein |
| ***Sobic.004G067850*** | 5529043..5532903 | 3861 | unknown |
| ***Sobic.004G067900*** | 5533571..5537014 | 3444 | similar to putative uncharacterized protein |
| ***Sobic.004G068000*** | 5537848..5538147 | 300 | unknown |
| ***Sobic.004G068100*** | 5538726..5545216 | 6491 | putative ankyrin repeat-containing protein |
| ***Sobic.004G068200*** | 5548222..5551449 | 3228 | similar to aspartic acid-rich protein |
| ***Sobic.004G068300*** | 5552126..5558631 | 6506 | similar to vacuolar H+-pyrophosphatase |
| ***Sobic.004G068350*** | 5565946..5571795 | 5850 | unknown |
| ***Sobic.004G068400*** | 5572016..5578763 | 6748 | similar to sucrose-phosphate synthase |
| ***Sobic.004G068500*** | 5581775..5585117 | 3343 | similar to putative uncharacterized protein |
| ***Sobic.004G068600*** | 5584655..5587070 | 2416 | cytochrome P450 CYP2 subfamily |
| ***Sobic.004G068700*** | 5601968..5604203 | 2236 | cytochrome P450 CYP2 subfamily |
| ***Sobic.004G068750*** | 5609811..5610704 | 894 | Unknown |
| ***Sobic.004G068800*** | 5612342..5614859 | 2518 | cytochrome P450 CYP2 subfamily |
| ***Sobic.004G068900*** | 5618766..5620768 | 2003 | cytochrome P450 CYP2 subfamily |
| ***Sobic.004G069000*** | 5623629..5627232 | 3604 | similar to putative uncharacterized protein |
| ***Sobic.004G069100*** | 5631446..5636643 | 5198 | F-box domain |
| ***Sobic.004G069200*** | 5637293..5640588 | 3296 | similar to receptor protein kinase |
| ***Sobic.004G069300*** | 5643529..5644768 | 1240 | unknown |
| ***Sobic.004G069400*** | 5643656..5647147 | 3492 | similar to mitochondrial transcription factor |
| ***Sobic.004G069500*** | 5647325..5649379 | 2055 | putative uncharacterized protein |
| ***Sobic.004G069600*** | 5650150..5651223 | 1074 | Ring zinc finger protein |
| ***Sobic.004G069700*** | 5655329..5657079 | 1751 | cytochrome P450 CYP2 subfamily |
| ***Sobic.004G069850*** | 5658787..5664962 | 6176 | putative cyclase |
| ***Sobic.004G070000*** | 5665682..5668760 | 3079 | similar to Metal-dependent hydrolase-like protein |
| ***Sobic.004G070100*** | 5669219..5673224 | 4006 | similar to putative uncharacterized protein |
| ***Sobic.004G070200*** | 5675175..5683769 | 8595 | mRNA cleavage and polyadenylation factor |
| ***Sobic.004G070300*** | 5683835..5684255 | 421 | unknown |
| ***Sobic.004G070400*** | 5701886..5703792 | 1907 | Myb/SANT-like DNA-binding domain |
| ***Sobic.004G070500*** | 5704083..5707546 | 3464 | inner membrane protein deda-related |
| ***Sobic.004G070600*** | 5709610..5713683 | 4074 | glycerophosphodiester phosphodiesterase-related |
| ***Sobic.004G070700*** | 5713684..5716369 | 2686 | putative uncharacterized protein |
| ***Sobic.004G070800*** | 5718507..5721635 | 3129 | putative uncharacterized protein |
| ***Sobic.004G070900*** | 5726939..5728555 | 1617 | similar to MYB transcription factor TaMYB1 |
| ***Sobic.004G071000*** | 5728604..5734362 | 5759 | similar to Cinnamyl alcohol dehydrogenase |
| ***Sobic.004G071100*** | 5743623..5746975 | 3353 | unknown |
| ***Sobic.004G071200*** | 5748572..5750859 | 2288 | similar to UDP-glucosyl transferase |
| ***Sobic.004G071300*** | 5761910..5766217 | 4308 | similar to putative uncharacterized protein |
| ***Sobic.004G071401*** | 5772315..5775534 2288 similar to UDP-glucosyl transferase | 3220 | elongation factor GTP binding domain |
| ***Sobic.004G071500*** | 5783527..5785921 | 2395 | similar to putative uncharacterized protein |
| ***Sobic.004G071600*** | 5789868..5790786 | 919 | putative uncharacterized protein |
| ***Sobic.004G071700*** | 5793660..5796548 | 2889 | similar to putative uncharacterized protein OJ1145_F01.17 |
| **SASb4581 marker** | 5819776..5819925 | NA | NA |

**Table S3 List of primers used for sequencing, qRT-PCR and constructs generation.**

| **Primer name** | **Forward sequence ( 5' to 3')** | **Reverse sequence (5' to 3')** |
| --- | --- | --- |
| **Primers used for PCR amplification and sequencing** | | |
| ***SbCAD2*_a** | TTTCTTTCCCGAAGGCTGAG | TTCGTGTTTGCACAAGGAGC |
| ***SbCAD2*-b** | CTATCTAGCTAACTGTGGCG | GCTATTTGACTTAAAAGGTC |
| ***SbCAD2*-c** | CTCCACTACTGCGAACTGAT | AGATAACGCTGAATGAACCT |
| ***SbCAD2*-d** | ACATGGGCGTGAAGGTGGCG | TCGGCGGTCTCGTCGATGCT |
| ***SbCAD2*-e** | ACCGCCGAGGTGCTCCAGTT | AGAACGCACAAAGCCCACAA |
| ***SbCAD2*-f** | AGATGGGGTACGTGAACGAG | AGTACGGAGTGTCGGAATCGT |
| **Promoter_a** | CCAAAAGATTGGGAGATGGA | TCTCCTCTTGGACTGGGACTG |
| **Promoter_b** | CGGAATGGATGATGTCAACT | TCTCCTCTTGGACTGGGACTG |
| **Promoter_c** | GGTGTTTTCTTGGCTGACCTA | TCTCCTCTTGGACTGGGACTG |
| **Primers used for qRT-PCR** | | |
| **CAD (*Sobic.004G071000*)** | AGGCCAACGTTGAGCAGTA | AGCGGGCTGTACACCGTTA |
| **Tubulin (*Sobic.002G350400*)** | AGCTCTCTGTGCCTGAAATCACCA | TTGAATCCAGTGGGGCACCAGTCC |
| **Primers used for generating constructs for transformation** | | |
| **PDA** | CGACTCTAGAGGATCAGTCTGCGGAACTTTACACG | GACCACCCGGGGATCTCTCCTCTTGGACTGGGACTG |
